# Atorvastatin Treatment of Rats with Ischemia-Reperfusion Injury Improves Adipose-Derived Mesenchymal Stem Cell Migration and Survival via the SDF-1α/CXCR-4 Axis

**DOI:** 10.1371/journal.pone.0079100

**Published:** 2013-12-02

**Authors:** Anping Cai, Ruofeng Qiu, Liwen Li, Dongdan Zheng, Yugang Dong, Danqing Yu, Yuli Huang, Shaoqi Rao, Yingling Zhou, Weiyi Mai

**Affiliations:** 1 Department of Cardiology, The First Affiliated Hospital of Sun Yat-sen University, Guangzhou, China; 2 Department of Cardiology, Guangdong Cardiovascular Institute, Guangdong General Hospital, Guangdong Academy of Medical Sciences, Guangzhou, China; 3 Department of Epidemiology and Health Statistics, Sun Yat-sen University, Guangzhou, China; University of Otago, New Zealand

## Abstract

**Background:**

Adipose-derived mesenchymal stem cells (ASCs) transplantation is a promising approach for myocardium repair. Promotion of ASCs migration and survival is the key for improving ASCs efficiency. SDF-1α is a critical factor responsible for ASCs migration and survival. Atorvastatin (Ator) is capable of up-regulating SDF-1α. Therefore, we're going to investigate whether ASCs migration and survival could be improved with atorvastatin.

**Methods:**

*In vitro* study, cardiomyocytes were subjected to anoxia-reoxygenation injury and subsequently divided into different groups: group blank control, Ator, Ator plus L-NAME (A+L-NAME) and Ator plus AMD3100 (A+AMD3100).When migration analysis completed, cardiomyocytes were used for subsequent analyses. *In vivo* study, rats underwent ischemia-reperfusion injury were assigned into different groups corresponding to *in vitro* protocols. ASCs were transplanted on the seventh day of atorvastatin therapy. Seven days later, the rates of migration, differentiation and apoptosis were evaluated.

**Results:**

Compared with other groups, ASCs migration *in vitro* was significantly improved in group Ator, which was dependent on SDF-1α/CXCR-4 coupling. Results of *in vivo* study were consistent with that of *in vitro* study, further supporting the notion that the efficacy of atorvastatin on ASCs migration improvement was related to SDF-1α/CXCR-4 axis. Higher vessel density in group Ator might be another mechanism responsible for migration improvement. Concomitantly, apoptosis was significantly reduced in group Ator, whereas no significant difference of differentiation was found.

**Conclusion:**

Migration and survival of ASCs could be improved by atorvastatin under ischemia-reperfusion injury, which were ascribed to SDF-1α/CXCR-4 axis.

## Introduction

Congestive heart failure (CHF) is still the leading cause of morbidity and mortality worldwide [1], [2]. Ischemic heart disease characterized by myocardial loss and cardiac remodeling is the major cause of CHF [3], [4]. During the past decades, a variety of strategies have been applied to improve outcome of ischemic heart disease, and one of the most promising and attractive approaches is adipose-derived mesenchymal stem cells (ASCs) transplantation [5]–[7]. ASCs are capable of conferring cardio-protective effects via multiple mechanisms such as trans-differentiation and secreting growth factors. Currently, the most commonly used methods for transplanting ASCs are intra-myocardial injection and peripheral intravenous infusion. In terms of less invasiveness and more clinical practice, peripheral infusion of ASCs must be more feasible and preferable [6], [8]. Nevertheless, with peripheral infusion, the amount of ASCs migrating to targeted tissue is far from enough to fully conduct repair and re-construction. Therefore, increasing the number of ASCs migrating to and retaining in cardiac tissues is the key prerequisite with respect to improve ASCs therapeutic efficiency.

Previously, a substantial body of studies has demonstrated that stromal-derived factor-1 alpha (SDF-1α, also known as CXCL12) and its specific receptor CXCR-4 played pivotal roles on ASCs migration and survival [9], [10]. Therefore, up-regulation of SDF-1α so as to promote SDF-1α/CXCR-4 coupling is essential and important for improving ASCs therapeutic efficiency. Literally, SDF-1α constitutively expresses in cardiac tissues and transiently and slightly increases after ischemic insult [11], [12]. Many strategies involving plasmid encoding for SDF-1α and direct intra-myocardial SDF-1α protein injection have been adopted to up-regulate SDF-1α expression. Recently, Feng and colleagues [13] reported that in the ischemia-reperfusion injury model, endothelial nitric oxide synthase (eNOS) over-expression in cardiac tissues promoted ASCs migration via SDF-1α up-regulation. Our previous study also showed that after 7 days of Atorvastatin (Ator) treatment, SDF-1α expression, dependent on eNOS/NO pathway, was also significantly increased in rats with acute myocardial infarction [14]. Taken together, we hypothesized that Atorvastatin, regarding its easily attainable virtue and attractive pleiotropic effects in terms of anti-apoptosis, anti-inflammation, anti-oxidation and pro-angiogenesis, might be used to substitute aforementioned complicated genetic approaches to govern ASCs migration and improve ASCs survival after peripheral infusion under acute ischemic injury setting. Furthermore, most of paramount importance, validation of this hypothesis would shed promising light on the future cardiac regenerative medicine with ASCs transplantation.

## Methods

### General experimental protocol and schematic

**Figure 1:**
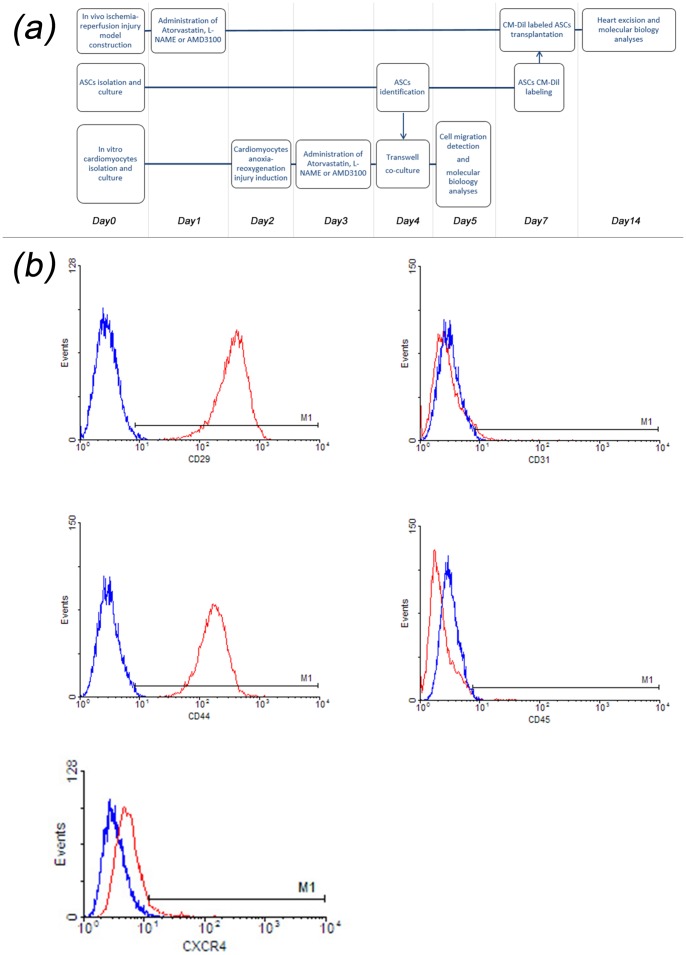
Study schematic and immuno-phenotype of ASCs. Panel (a) Schematic of the *in vivo* and *in vitro* experiment. Panel (b) CD29, CD31, CD44 and CD45 were detected by flow cytometry. Results showed that the fourth passage ASCs were largely positive for CD29 (99.80±0.10%) and CD44 (99.60±0.20%), and only minority of ASCs were positive for CD31 (0.30±0.10%) and CD45 (0.45±0.10%). The expression of CXCR4 on ASCs was 9.96±0.07%.

As present in Figure 1 panel (a), for *in vitro* evaluation, ASCs and neonatal cardiomyocytes were previously prepared. After anoxia-reoxygenation injury was produced in cardiomyocytes on day 2, different treated regimens were implemented. Twenty-four hours later, ASCs were placed in upper chambers of the Trans-well system for migration analysis. Afterward, cardiomyocytes were used for subsequent analyses. For *in vivo* evaluation, on day 0, ischemia-reperfusion injury was produced in rat, and 24 hours later different treated regimens were administered for 14 days (from day 1 to day 14). On day 7, labeled ASCs were infused via caudal vein, and 7 days later (on day 14) hearts were harvested for following analyses. Three independent experiments were performed in duplicate.

### ASCs isolation, culture and flow-cytometry analysis

Male Sprague-Dawley (SD) rats weighing 100–120 g were obtained from Experimental Animal Center of Sun Yat-sen University, Guangzhou, China. The study was approved by Ethic Committee of Sun Yat-sen University (Permit Number: IACUC-2013-0303). Protocols of isolation and culture of ASCs were in accordance to our previous study [7]. Adipose tissues were isolated from bilateral inguinal regions of SD rats, washed with D-Hank's solution, minced into small pieces, digested with 0.1% type I collagenase solution (Sigma, St. Louis, US) at 37°C for 1 hour, and then inactivated with Dulbecco's modified Eagle's medium (DMEM)/F12 (Hyclon, Logan, USA) supplemented with 10% fetal bovine serum (FBS, Gibco). The cells were seeded at a density of 2×10^6^ cells/mL in DMEM/F12, supplemented with 10% FBS, in T-75 tissue culture flasks (Corning, New York, USA) in humidified air with 5% CO_2_ at 37°C. When the cells reached 80% confluence, adherent cells were digested with 0.125% trypsin and 0.01% EDTA and then reseeded at a 1∶3 dilution. For immuno-phenotype and CXCR4 expression analysis, the fourth passage ASCs were labeled with fluorescent anti-rat antibodies CD29-FITC (BD pharmingen, San Diego, CA, 555005, 1∶100), CD31-PE (BD pharmingen, San Diego, CA, 555027, 1∶100), CD44-PE (Abcam, Cambridge, UK, ab23396, 1∶100), CD45-FITC (BD pharmingen, San Diego, CA, 554877, 1∶100) and CXCR4-FITC (Abcam, Cambridge, UK, ab2074, 1∶50) and finally underwent flow-cytometry evaluation.

### In vitro ASCs migration analysis

The study was approved by Ethic Committee of Sun Yat-sen University (Permit Number: IACUC-2013-0303). Cardiomyocytes were isolated from neonatal SD rats according to our previous study [7] and seeded at a density of 1×10^6^ cells in the lower chambers of Trans-well system with 8.0 µm pore polycarbonate membrane insert (Corning, New York, USA, #3428). Forty-eight hours later, *in vitro* anoxia-reoxygenation (AR) injury was produced by placing cardiomyocytes in a hypoxia incubator filled with 5% CO_2_ and 95% N_2_ at 37°C for 60 minutes, and then re-oxygenation with 5% CO_2_ and 95% O_2_ for 60 minutes. Cardiomyocytes subjected to AR injury were divided into 4 groups as follow: group blank control, Ator, Ator plus NG-Nitro-L-arginine Methyl Ester (A+L-NAME) and Ator plus AMD3100 (A+AMD3100). Cardiomyocytes without AR injury were used as sham operated group. In group Ator, A+L-NAME and A+AMD3100, Atorvastatin (Sigma, St. Louis, MO, PZ0001, dissolved in dimethyl-sulfoxide, DMSO, working concentration 10 µmol/L) was added into culture medium, while same volume of DMSO was added into group blank control. L-NAME (100 µmol/L, dissolved in PBS, Sigma, St. Louis, MO, N5751) or AMD3100 (10 mg/L, dissolved in PBS, Sigma, St. Louis, MO, A5602)were concomitantly added into group A+L-NAME or A+AMD3100 respectively, and same volume of PBS was added into group blank control and Ator. Twenty-four hours later, 1×10^5^ ASCs were seeded at the upper chambers of the Trans-well system for *in vitro* migration analysis. Twelve hours later, the inserts were fixed with 11% glutaraldehyde (Sigma, St. Louis, MO, G6257), stained with crystal violet solution (Sigma, St. Louis, MO, V5265), washed with double distilled water, and examined under microscope. The number of migrated cells in each insert was counted at 10 random high power fields (200× magnification).

### In vivo ischemia-reperfusion (IR) injury and atorvastatin administration

Male SD rats weighing 200–220 g were obtained from Experimental Animal Center of Sun Yat-sen University, Guangzhou, China. The study was approved by Ethic Committee of Sun Yat-sen University (Permit Number: IACUC-2013-0303). All surgery was performed under chloral hydrate anesthesia, and all efforts were made to minimize suffering. All animals received humane care in compliance with the Guide for the Care and Use of Laboratory Animals of the Institute of Laboratory Animal Resources, National Research Council.

IR injury in rats was produced by transient left anterior coronary artery occlusion for 60 minutes and then followed by reperfusion. Thirty-six survived rats were randomly and evenly assigned into four groups as follow: group blank control, Atorvastatin (Ator), Atorvastatin plus L-NAME (A+L-NAME), and Atorvastatin plus AMD3100 (A+AMD3100). Nine rats served as sham operated group, in which similar procedures were performed while without coronary artery ligation. Twenty-four hours later (on day 1), atorvastatin (10 mg/Kg/d body weight, reconstituted in normal saline) was given by gavage once daily to group Ator, A+L-NAME and A+AMD3100, and same volume of saline was given to group blank control and sham operated. Eight hours later, L-NAME (40 mg/Kg/d body weight, dissolved in normal saline) was given by gavage to group A+L-NAME, and same volume of saline was given to other groups. AMD3100 (5 mg/Kg/d body weight, dissolved in normal saline) was given to group A+AMD3100 by subcutaneous injection, and same volume of saline was subcutaneously injected to other groups. The therapeutic course was 14 days.

### Labeling and transplantation of ASCs, and evaluation of migration, differentiation and apoptosis in vivo

The fourth passage ASCs were stained with CellTracker™ CM-DiI (Invitrogen, Carlsbad, California, C7000). All the procedures were performed according to manufacturer's instruction. On day 7, 1×10^7^ labeled ASCs were transplanted to each rat by caudal vein infusion. On day 14, hearts were harvested, placed in embedding medium (Tissue-Tek, Sakura, Japan) and underwent transverse section for immuno-fluorescent analyses. Sections were fixed in 4% paraformaldehyde for 30 minutes, rinsed with PBS, and blocked with 0.1% Tween in Phosphate Buffer Saline (PBS-T) containing 1% bovine serum albumin (BSA) at room temperature for 1 hour. Afterward, sections were incubated with mouse anti-rat Troponin-I antibody (Santa Cruz, CA, sc-133177, 1∶50) at 4°C overnight, washed with PBS, incubated with goat anti-mouse Alexa Fluor 488 secondary antibody (Cell Signaling Technology, Danvers, MA, #4408, 1∶500), followed by DAPI (Roche, Mannheim, Germany, 10236276001, 1 µg/ml) staining, and examined under fluorescence microscope(200× magnification). ASCs were stained with red color and cardiomyocytes were green. For migration evaluation, the number of ASCs in 10 random fields of peri-infracted region was counted in each section. For evaluation of differentiation, 100 ASCs were counted in each section, and Troponin-I positive ASCs were regarded as differentiating towards cardiomyocytes. Differentiation rate was calculated as the number of Troponin-I positive ASCs divided by 100. For apoptosis analysis, TdT-mediated dUTP nick-end labeling (TUNEL) (Roche, Mannheim, Germany, 11772465001) was performed according to the manufacture's instruction. Sections were examined under fluorescence microscope at 400× magnification. Nuclei were stained with DAPI and presented as blue color while apoptotic nuclei were green, and transplanted ASCs presented with red color. One hundred ASCs nuclei were counted in each section, and the apoptotic index was calculated with the formula: the number of TUNEL positive ASCs nuclei divided by 100.

### Assessment of vessel density in peri-infarcted region

On day 14, immuno-staining was performed to evaluate vessel density in peri-infarcted areas. Sections were fixed in 4% paraformaldehyde, rinsed with PBS, and blocked with 0.1% PBS-T containing 1% BSA. After co-incubated with rabbit anti-rat CD31(Santa Cruz, CA, sc-1506R, 1∶50) and mouse anti-rat α-actin antibody (Sigma, St. Louis, MO,A7811, 1∶1000) at 4°C overnight, sections were washed with PBS, and co-incubated with goat anti-rabbit Alexa Fluor 488 (Cell Signaling Technology, Danvers, MA, #4412, 1∶500) and donkey anti-mouse NL557 secondary antibody (R&D systems, Minneapolis, MN, NL007, 1∶100). Cardiomyocytes were stained with red color and vascular endothelial cells were green under fluorescence microscope at 200× magnification. The number of vessels in 10 random fields of peri-infracted region in each section was counted.

### Analysis of eNOS and p-eNOS expression by Western blot

Expression of eNOS and p-eNOS was analyzed by Western blot. For *in vivo* study, protein was extracted from infarcted and peri-infarcted regions of myocardium. For *in vitro* study, protein was extracted from cardiomyocytes when ASCs migration analysis was completed. Cardiac tissues and cardiomyocytes were homogenized and lysed with 500 µL of lysis buffer (50 mM Tris-HCl, pH 7.5; 5 mM EDTA; 250 mM NaCl; and 0.1% Triton X-100) containing 20 µL (10 mg/ml) of protease inhibitors Phenylmethanesulfonyl Fluoride (PMSF), respectively. Concentration of protein was measured by Bicinchoninic acid (BCA) method. Sample was electrophoresed on 8% SDS-polyacrylamide gel and then transferred onto Polyvinylidene Fluoride (PVDF) membranes. After blocking with 0.1% Tween in Tris-buffered saline (TBS-T) containing 5% BSA at room temperature for 1 hour, membranes were incubated at 4°C overnight with rabbit anti-rat eNOS and p-eNOS antibodies (Cell Signaling Technology, Danvers, MA, #9586 and #9570, 1∶1000). The membranes were washed three times with TBS-T and incubated with goat anti-rabbit IgG HRP-conjugated secondary antibody (Cell Signaling Technology, Danvers, MA, #7074, 1∶3000) at room temperature for 1 hour. GAPDH was used as a loading control. Interested protein was detected by chemiluminescence, and optical density (OD) of each group was measured in grey scale images with Volume Contour method and the data was normalized to GAPDH.

### Measurement of NO production

Total nitric oxide (NO) was evaluated by nitrite reductase method using Total Nitric Oxide Kit (Beyotime, Haimen, China, S0023). All the procedures were performed according to the manufacturer's instruction. For *in vivo* study, protein was extracted from infarcted and peri-infarcted regions of myocardium. For *in vitro* study, protein was extracted from cardiomyocytes when ASCs migration analysis was completed. Cardiac tissues and cardiomyocytes were homogenized and lysed with 500 µL of lysis buffer (50 mM Tris-HCl, pH 7.5; 5 mM EDTA; 250 mM NaCl; and 0.1% Triton X-100) containing 20 µL (10 mg/ml) of protease inhibitors Phenyl-methane-sulfonyl Fluoride (PMSF), respectively. Concentration of protein was measured by Bicinchoninic acid (BCA) method. After quantified by BCA method, protein concentration was adjusted to 10 mg/ml. Potassium Nitrite (KNO_2_) supplied within the Total Nitric Oxide Kit was used to figure out standard curve. Optical density of each sample was detected by Enzyme-labeling measuring instrument at a wavelength of 550 nm, and the concentration of NO was figured out upon the standard curve.

### Evaluation of SDF-1α expression

Quantitative immunoassay was used for evaluating SDF-1α expression. For *in vivo* study, protein was extracted from infarcted and peri-infarcted regions of myocardium. For *in vitro* study, protein was extracted from cardiomyocytes when ASCs migration analysis was completed. Cardiac tissues and cardiomyocytes were homogenized and lysed with 500 µL of lysis buffer (50 mM Tris-HCl, pH 7.5; 5 mM EDTA; 250 mM NaCl; and 0.1% Triton X-100) containing 20 µL (10 mg/ml) of protease inhibitors PMSF, respectively. Concentration of protein was measured by Bicinchoninic acid (BCA) method. All procedures were performed according to manufacturer's instructions (R&D Systems, Minneapolis, MN). Protein concentration was adjusted to 10 mg/ml. Recombinant murine SDF-1α supplied within the kit was used to figure out standard curve. Optical density of each sample was detected by Enzyme-labeling measuring instrument at a wavelength of 460 nm, and the concentration of SDF-1α was figured out upon the standard curve.

### Statistical analysis

All continuous variables were expressed as mean±SD, and analyses were performed with SPSS software, version 16.0 (SPSS Science, Chicago, IL, USA). Statistical significance among groups was evaluated with One Way ANOVA (post hoc LSD-t), and a value of *P*<0.05 was considered statistically significant.

## Results

### Immuno-phenotype and Expression of CXCR4 of ASCs

As shown in Figure 1 panel (b), a vast majority of the fourth passage ASCs were positive for ASCs specific markers CD29 and CD44, while only minority of ASCs were positive for endothelial cells marker CD31 and hematopoietic cells marker CD45. The expressions of CXCR4 on ASCs were 9.96%±0.07% which was similar to previous reports [15], [16].

### Atorvastatin increased SDF-1α expression in vivo under ischemia-reperfusion injury

**Figure 2:**
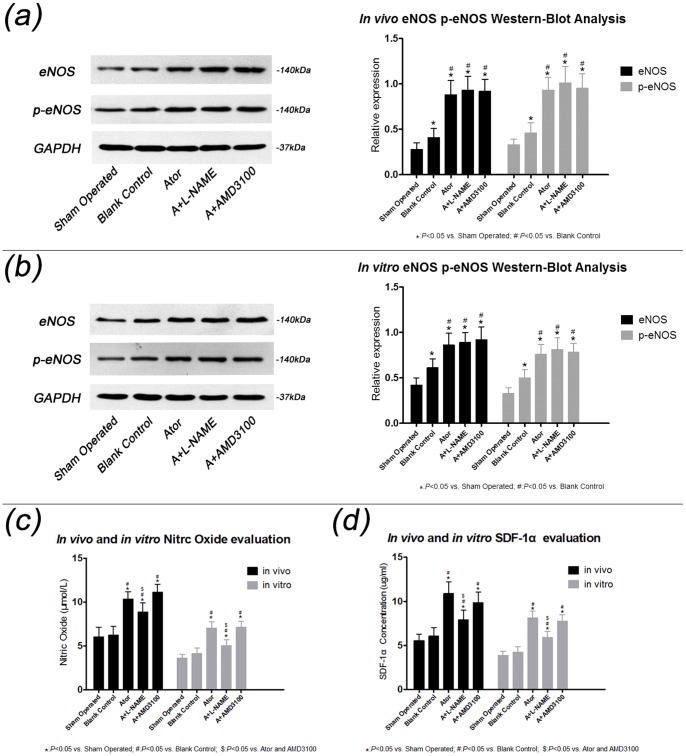
Comparisons of eNOS, p-eNOS, NO and SDF-1α levels in each group. Panel (a) Western-blot analysis showed that a significant increment of eNOS expression in group Ator, A+L-NAME and A+AMD3100 for *in vivo* study when compared with blank control. (sham operated: 0.28±0.07, blank control: 0.41±0.10*, Ator: 0.88±0.16*#, A+L-NAME: 0.93±0.15*#, A+AMD3100: 0.92±0.13*#). Similar changes were found in p-eNOS expression among each group (sham operated: 0.33±0.06, blank control: 0.46±0.11*, Ator: 0.93±0.14*#, A+L-NAME: 1.01±0.18*#, A+AMD3100: 0.95±0.16*#). Panel (b) Analyses of eNOS expression by Western-blot for *in vitro* study showed that as compared with group blank control, eNOS was significantly increased in group Ator, A+L-NAME and A+AMD3100. (sham operated: 0.42±0.08, blank control: 0.61±0.10*, Ator: 0.86±0.13*#, A+L-NAME: 0.89±0.11*#, A+AMD3100: 0.92±0.14*#). Similar changes were found in p-eNOS expression among each group (sham operated: 0.33±0.06, blank control: 0.50±0.09*, Ator: 0.76±0.11*#, A+L-NAME: 0.81±0.13*#, A+AMD3100: 0.78±0.10*#). Panel(c) When compared with group blank control, the NO productions *in vitro* and *in vivo* were identically and significantly increased in group Ator and A+AMD3100, whereas was abolished in group A+L-NAME. (*In vivo* study, sham operated: 6.03±1.10, blank control: 6.22±0.98, Ator: 10.31±0.86*#, A+L-NAME: 8.86±1.03*#$, A+AMD3100: 11.10±0.92*#) and (*in vitro* study, sham operated: 3.62±0.42, blank control: 4.11±0.66, Ator: 7.03±0.71*#, A+L-NAME: 5.02±0.69*#$, A+AMD3100: 7.11±0.70*#). Panel (d) As compared with group blank control, the SDF-1α expressions *in vitro* and *in vivo* was profoundly increased with atorvastatin therapy; however, the effects were reduced in group A+L-NAME. (*In vivo* study, sham operated: 5.53±0.73, blank control: 6.05±0.95, Ator: 10.88±1.33*#, A+L-NAME: 7.92±1.09*#$, A+AMD3100: 9.86±1.21*#) and (*in vitro* study, sham operated: 3.84±0.47, blank control: 4.21±0.65, Ator: 8.12±0.77*#, A+L-NAME: 5.88±0.70*#$, A+AMD3100: 7.76±0.73*#).

As shown in Figure 2 panel (a), as compared with group blank control, eNOS and p-eNOS were significantly increased in 3 atorvastatin treated groups, indicating that atorvastatin enhanced eNOS and p-eNOS expression under IR injury. Similar changes were also found in *in vitro* analysis. However, as shown in Figure 2 panel (c and d), compared with group Ator and group A+AMD3100, SDF-1α upregulation was significantly diminished in group A+L-NAME when NO production was reduced by L-NAME addition, suggesting that SDF-1α upregulation by atorvastatin was at least partially associated with the upregulation of eNOS/NO under IR.

### Atorvastatin increased eNOS/p-eNOS expression of ASCs in vitro under anoxia-reoxygenation injury

As shown in Figure 2 panel (b), in *in vitro* analysis of ASCs under anoxia-reoxygenation injury, as compared with group blank control, eNOS was significantly increased in group Ator, A+L-NAME and A+AMD3100. Similar changes were found in p-eNOS expression among each group.

### Comparisons of ASCs migration and differentiation in vivo

**Figure 3:**
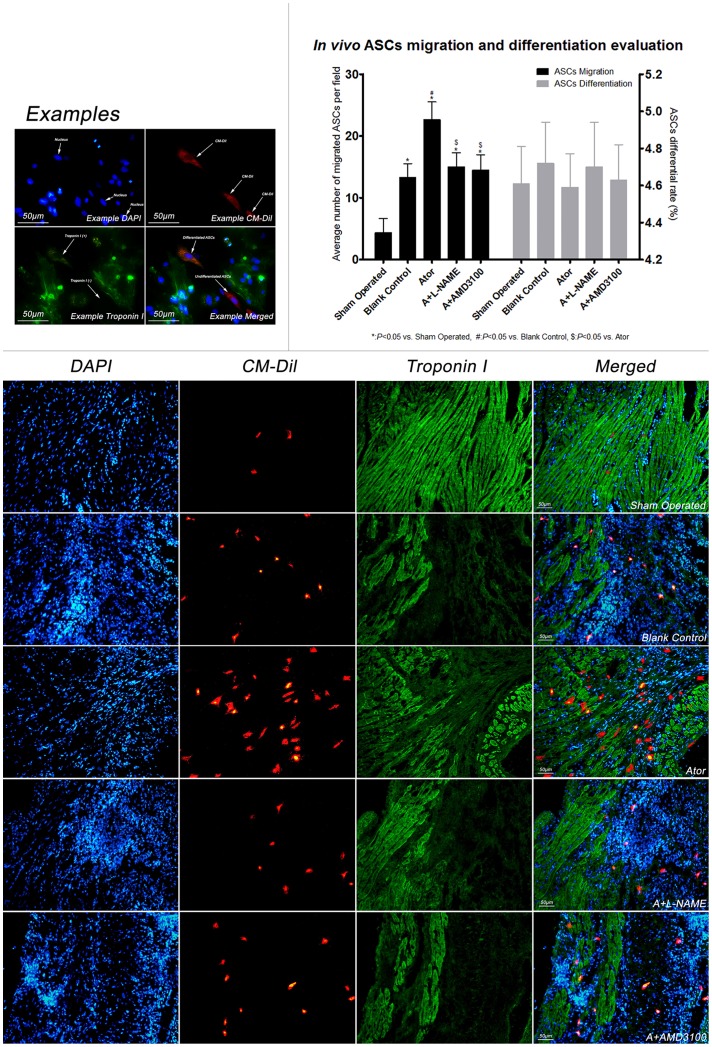
Evaluation of ASCs migration and differentiation *in vivo*. Seven days after ASCs transplantation, immuno-staining analysis showed that in comparison of group blank control, the number of ASCs migrated in peri-infarcted areas was significantly increased in group Ator, however, the efficacy with atorvastatin therapy was abolished when SDF-1α up-regulation was diminished or SDF-1α/CXCR-4 coupling was blockage as shown in group A+L-NAME and A+AMD3100. (sham operated: 4.4±2.3, blank control: 13.3±2.2*, Ator: 22.6±2.9*#, A+L-NAME: 15.0±2.3*$, A+AMD3100: 14.5±2.5*$, per high power field). No significant difference was found in ASCs differentiation among each group (sham operated: 4.6±0.2%, blank control: 4.7±0.2%, Ator: 4.5±0.2%, A+L-NAME: 4.7±0.2%, A+AMD3100: 4.6±0.2%). (Scale Bar: 50 µm).

As shown in Figure 3, immuno-staining showed that after 14 days of atorvastatin treatment, the number of ASCs migrating to and retaining in peri-infarcted areas was significantly higher in group Ator than the other groups, suggesting that both SDF-1α up-regulation and SDF-1α/CXCR-4 coupling are critical for improvement of ASCs migration. Furthermore, differentiation rate was concomitantly evaluated, where no significant difference among each group was found.

### In vitro ASCs migration was improved by atorvastatin treatment

**Figure 4:**
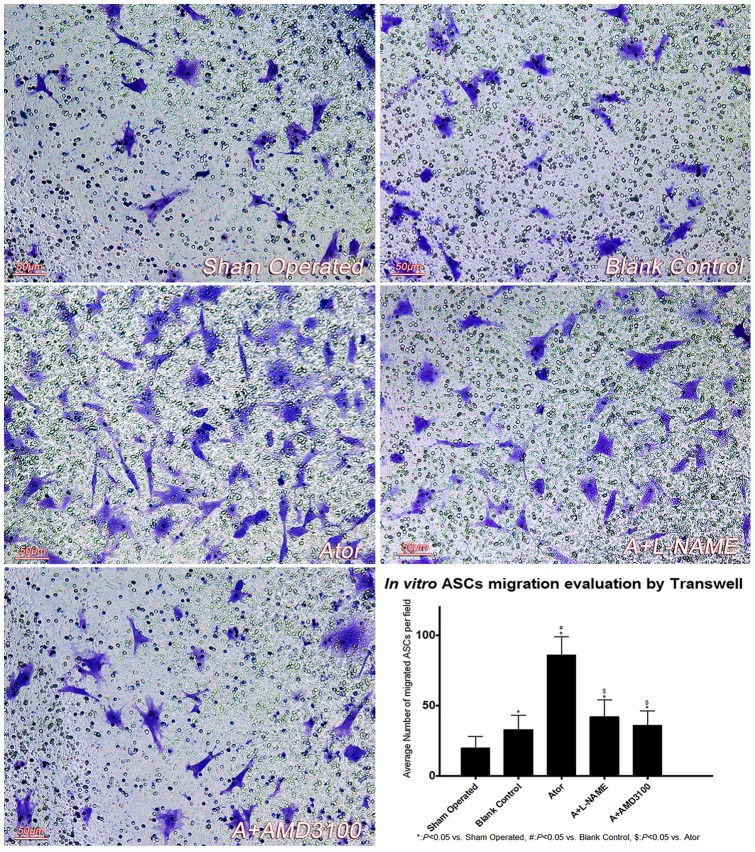
ASCs *in vitro* migration analysis. ASCs *In vitro* migration was performed in a Trans-well system. Atorvastatin treatment significantly promoted ASCs migration under anoxia-reoxygenation injury, which was dependent on both SDF-1α up-regulation and SDF-1α/CXCR-4 coupling. (sham operated: 20.1±8.3, blank control: 33.6±10.2*, Ator: 86.7±13.1*#, A+L-NAME: 42.0±12.9*$, A+AMD3100: 36.8±10.2*$, per high power field). (Scale Bar: 50 µm).

The outcome of *in vitro* ASCs migration analyses was consistent with *in vivo* study, as revealed by the efficacy of atorvastatin (group Ator) on ASCs migration improvement was mediated by SDF-1α/CXCR-4 axis, since either SDF-1α reduction (group A+L-NAME) or SDF-1α/CXCR-4 coupling blockage (group A+AMD3100) profoundly reduced ASCs migration (See Figure 4).

### Enhancement of vessel density by atorvastatin treatment

**Figure 5:**
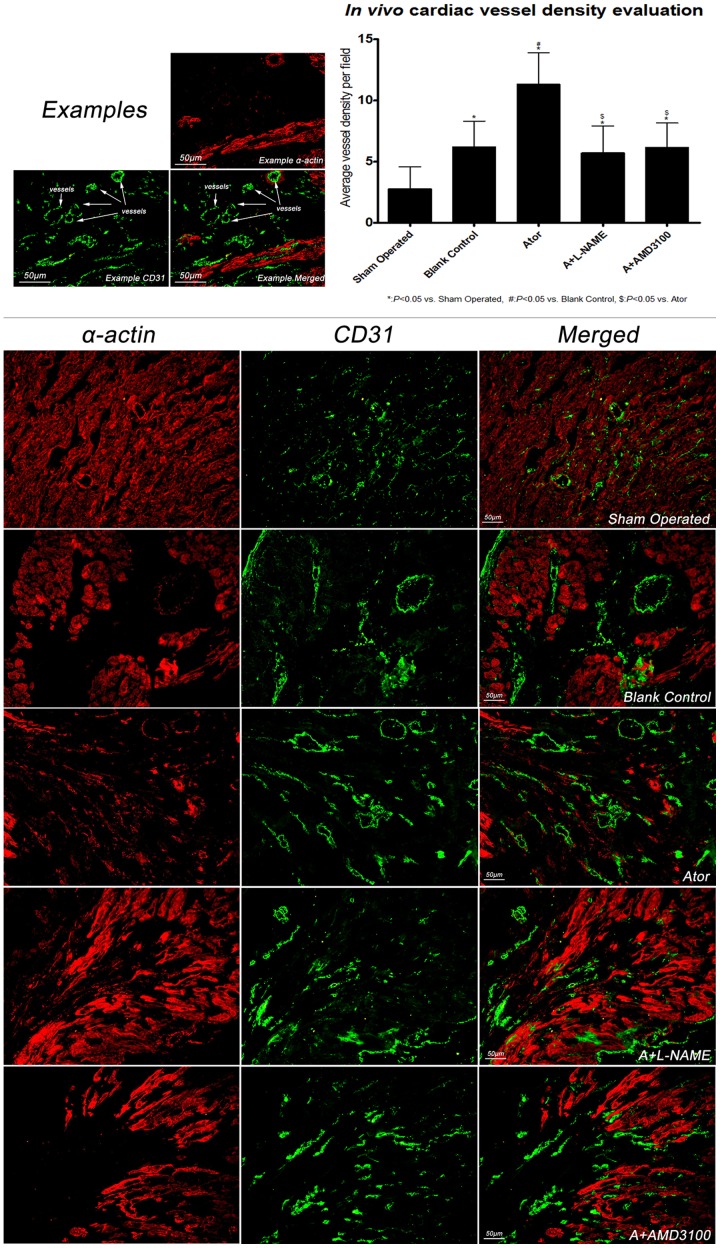
Assessment of cardiac vessel density. Vessel density in peri-infarcted area was significantly higher in group Ator than the other groups under immuno-staining evaluation (sham operated: 2.8±1.8, blank control: 6.2±2.1*, Ator: 11.3±2.6*#, A+L-NAME: 5.7±2.2*$, A+AMD3100: 6.1±2.0*$, per high power field). (Scale Bar: 50 µm).

As shown in Figure 5, vessel density in peri-infarcted areas was evaluated on day 14. Comparing with group blank control, vessel density was higher in group Ator. Nevertheless, as compared with group Ator, vessel density in group A+L-NAME and A+AMD3100 was diminished, indicating that the effect of atorvastatin on angiogenesis was at least partially attributed to eNOS/NO and SDF-1α/CXCR-4 pathway.

### ASCs apoptosis was attenuated by atorvastatin treatment in vivo

**Figure 6:**
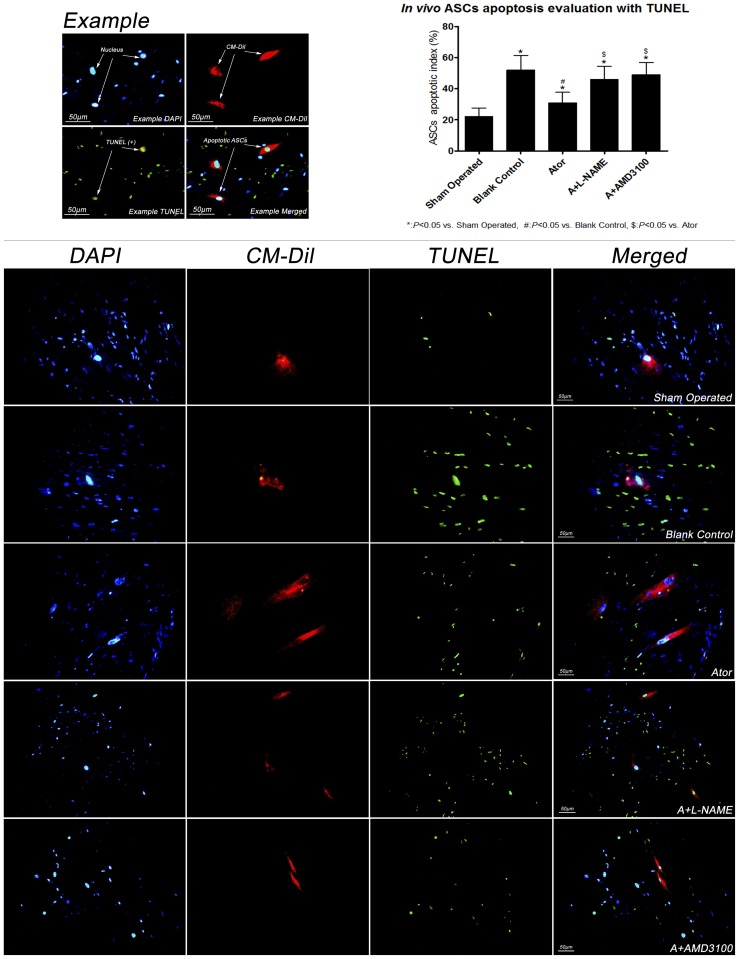
Comparison of ASCs apoptotic index *in vivo.* * In vivo* apoptotic index was detected by TUNEL, and the results showed that compared with group blank control, atorvastatin therapy significantly reduced apoptotic index of ASCs, nevertheless, the efficacy was offset when either L-NAME or AMD3100 was added. (sham operated: 22.6±5.5, blank control: 52.1±9.4*, Ator: 31.3±6.7*#, A+L-NAME: 46.0±8.3*$, A+AMD3100: 49.7±7.8*$, per high power field). (Scale Bar: 50 µm).

As shown in Figure 6, apoptotic index of ASCs *in vivo* was evaluated by TUNEL. Comparing with group blank control, ASCs apoptosis in group Ator was significantly reduced. The addition of L-NAME or AMD3100 attenuated the anti-apoptotic effect of atorvastatin, suggesting that the anti-apoptotic effect conferred by atorvastatin was related to NO and SDF-1α up-regulation and SDF-1α/CXCR-4 coupling.

## Discussion

Our present study showed that compared with blank control group, atorvastatin treatment significantly promoted ASCs oriented migration and reduced ASCs apoptosis in rat with ischemia-reperfusion injury. The mechanisms were at least partially related to SDF-1α up-regulation and SDF-1α/CXCR-4 coupling as evidenced by that when either SDF-1α expression was reduced in group A+L-NAME or SDF-1α/CXCR-4 coupling was blocked in group A+AMD3100, those beneficial effects conferred by atorvastatin treatment were profoundly abrogated. Furthermore, the increased number of transplanted ASCs observed in cardiac tissue from Ator group could not only be due to increased migration, but also may be due to better cell survival in the ischemic tissue. Notably, data from migration analysis in vitro further supported the notion that the improved effects of atorvastatin therapy on ASCs migration were mediated by SDF-1α up-regulation and SDF-1α/CXCR-4 coupling under anoxia-reoxygenation condition.

Congestive heart failure, largely owing to ischemic heart disease, is a progressive disease with poor prognosis, despite optimal medication and device approaches are applied. Heart transplantation is hampered by lack of donor and other ethical and moral issues. Encouragingly, mesenchymal stem cells characterized by multiple differentiation potential and easy accessibility are recognized as an attractive and promising approach for myocardium repair and re-construction [17], [18]. By virtue of less invasiveness and more clinical practice, peripheral intravenous infusion of ASCs seems to be more preferable than other methods such as intra-cardiac injection. Nevertheless, only a minority of infused cells could migrate to and survive in ischemic cardiac tissue, which significantly hinders the technique to be applied in cardiac regenerative medicine with respect to ASCs implantation. Accordingly, SDF-1α/CXCR-4 axis plays an important role on ASCs migration and survival. Therefore, many genetic approaches have been investigated to manipulate local SDF-1α over-expression after ischemic insult and the outcomes are quite striking although those methods seems less practical and infeasible under present circumstance. To our best knowledge, this was for the first study revealing that atorvastatin, a commonly used medication, was able to enhance SDF-1α expression in cardiac tissues after ischemia-reperfusion injury, thereby subsequently resulting in improvement of ASCs oriented migration. We deduce that at least one of the mechanisms involved in atorvastatin enhancingSDF-1α expression was dependent on eNOS/NO pathway, which was consistent with previous study [13]. Furthermore, *in vitro* migration analysis substantially demonstrated that improvement of ASCs migration by Atorvastatin was also attributed to the increase of SDF-1α expression in cardiomyocytes with anoxia-reoxygenation injury. On the other hand, increase of vessels density in peri-infarcted regions might be another potential mechanism by which atorvastatin promoted ASCs oriented migration *in vivo*, since more vessels in peri-infarcted region could result in more blood with ASCs flowing into cardiac tissues thereby increasing regional cells retaining. As is well known, one of the pleiotropic effects of atorvastatin is pro-angiogenesis, which is considered to be associating with endothelial progenitor cells mobilization. Intriguingly, some studies reported that SDF-1α up-regulation in ischemic cardiac tissues could also increase vessel density which might be partially due to the enhancement of SDF-1α coupling with circulating CXCR-4 positive stem cells [19], [20]. Collectively, we considered that the underlying mechanisms by which atorvastatin therapy improved ASCs migration were both related to SDF-1α up-regulation in cardiomyocytes under ischemic insult and the increase of peri-infarcted vessels density. Moreover, with respect to the advantages of atorvastatin treatment in terms of easily attainable virtue, safely use in human, and has beneficial pleiotropic effects, we believed that with the use of atorvastatin to enhance local SDF-1α expression was superior to the aforementioned genetic approaches which necessitate complicated preparation, require invasive procedure for local injection, only have been tested in animal models, and are without statins' pleiotropic effects.

Improvement of transplanted cells survival in ischemic cardiac tissue is another critical issue needed to be resolved. As is well known that cardiac microenvironment is unfavorable for transplanted cells to survive after acute ischemic insult, which is largely due to local inflammation and oxidation, and activation of pro-apoptosis signaling pathway. Our previous study showed that atorvastatin could ameliorate cardiac hostile milieu by its pleiotropic effects, and consequently improved the number of transplanted cells in cardiac tissues when compared with control group [7]. Furthermore, SDF-1α/CXCR-4 coupling could also activate pro-survival signaling pathways, which finally contribute to ASCs apoptosis reduction [21], [22]. Our previous study also showed that 7 days of atorvastatin treatment could increase Bcl-2/Bax ratio and p-Akt expression in rat with acute ischemic insult [14]. Therefore, besides other pleiotropic effects, SDF-1α up-regulation under ischemia-reperfusion injury might be another mechanism by which atorvastatin improved ASCs survival.

Unexpectedly, although migration and survival rates were significantly improved in group Ator when compared with blank control, the differentiation rate was without any improvement, and no significant difference among each group was found.

## Conclusion

Our study presented for the first time that when compared with control group, atorvastatin could potentially increase SDF-1α expression after ischemia-reperfusion injury, thereby consequently promoting ASCs migration and survival. Consideration of procedure-friendly method and easy application in clinical practice, peripheral intravenous infusion of ASCs, likewise hematopoietic stem cells transplantation in leukemia patients, could be another encouraging and promising approach for myocardium regenerative medicine, especially when an adjunctive tool could be “off-the shelf” used for facilitating transplanted cells migration and survival.
